# 13-Methylberberine improves endothelial dysfunction by inhibiting NLRP3 inflammasome activation via autophagy induction in human umbilical vein endothelial cells

**DOI:** 10.1186/s13020-020-0286-1

**Published:** 2020-01-22

**Authors:** Zhihua Peng, Hong Zhan, Yijia Shao, Yan Xiong, Lijin Zeng, Cong Zhang, Zhihao Liu, Zhenhua Huang, Huanxing Su, Zhen Yang

**Affiliations:** 1 Division of Emergency Medicine, Department of Emergency Intensive Care Unit, The First Affiliated Hospital, Sun Yat-Sen University, Guangzhou, 510080 China; 2 Department of Cardiology, The First Affiliated Hospital, Sun Yat-Sen University, Guangzhou, 510080 Guangdong China; 3 Key Laboratory on Assisted Circulation, Ministry of Health, The First Affiliated Hospital, Sun Yat-Sen University, Guangzhou, 510080 China; 4 State Key Laboratory of Quality Research in Chinese Medicine, Institute of Chinese Medical Sciences, University of Macau, Macao, China

**Keywords:** 13-Methylberberine, Atherosclerosis, Anti-inflammatory, Autophagy inducer, NLRP3 inflammasome

## Abstract

**Background:**

Atherosclerosis, the underlying cause of the majority of cardiovascular diseases, is a lipid-driven, inflammatory disease of the large arteries. Atherosclerotic cardiovascular disease (ASCVD) threatens human lives due to high morbidity and mortality. Many studies have demonstrated that atherosclerosis is accelerated via activation of the NLRP3 inflammasome. The NLRP3 inflammasome plays a critical role in the development of vascular inflammation and atherosclerosis. In atherosclerotic plaques, excessive generation of reactive oxygen species (ROS) activates the NLRP3 inflammasome. 13-Methylberberine (13-MB) is a newly synthesized compound used in traditional Chinese medicine that has outstanding antibacterial, antitumor, and antiobesity activities, especially anti-inflammatory activity. However, the role of 13-MB in atherosclerosis needs to be explored.

**Methods:**

CCK-8 assays and flow cytometry were conducted to determine the cell viability and apoptotic profiles of human umbilical vein endothelial cells (HUVECs) treated with 13-MB. Carboxy-DCFH-DA and JC-10 assays were used to measure ROS and determine mitochondrial membrane potential. Western blot analysis was performed to investigate proteins that are associated with the NLRP3 inflammasome and autophagy. ELISA was used to detect and quantify inflammatory cytokines related to the NLRP3 inflammasome. Transfection and confocal microscopy were conducted to observe autophagy.

**Results:**

Pretreatment with 13-MB markedly reduced cytotoxicity and apoptosis, as well as intracellular ROS production, in H_2_O_2_-induced HUVECs. Moreover, 13-MB showed a protective effect in maintaining mitochondrial membrane potential. 13-MB also suppressed NLRP3 inflammasome activation and promoted autophagy induction in HUVECs.

**Conclusion:**

13-MB exerts cytoprotective effects in an H_2_O_2_-induced cell injury model by inhibiting NLRP3 inflammasome activation via autophagy induction in HUVECs. These anti-inflammatory and autophagy induction activities may provide valuable evidence for further investigating the potential role of 13-MB in atherosclerosis.

## Background

Atherosclerosis is the most common cause of the underlying pathology of cardiovascular disease. It is characterized as a lipid-driven, chronic inflammatory disease of the large arteries, leading to high morbidity and mortality worldwide [1, 2]. Vascular endothelial inflammation has an overwhelming role in atherosclerosis [3, 4]. The NLRP3 inflammasome is involved in the chronic inflammation that underlies atherogenesis in vessel walls [5]. There is a link between inflammation and lipid metabolism. Crystalline cholesterol, oxidized low-density lipoprotein (ox-LDL), oxidative stress and mitochondrial dysfunction are implicated as important stimuli of vascular endothelial inflammation in atherosclerosis [6]. Reactive oxygen species (ROS) play an essential role in NLRP3 inflammasome activation in atherosclerosis [7]. Moreover, autophagy and inflammation are known to interact on multiple levels [8]. Accumulating evidence suggests that autophagy is stimulated by oxidized lipids, inflammation, and metabolic stress conditions in atherosclerotic plaques. Autophagy is antiapoptotic and contributes to cell survival in adverse environments [9, 10]. Interestingly, basal autophagy can be intensified by specific drugs. Because atherosclerosis is an inflammatory disorder of the arterial intima, pharmacological anti-inflammatory approaches may be developed to stabilize vulnerable, rupture-prone lesions through autophagy induction [11].

13-Methylberberine (13-MB) is a newly synthesized compound used in traditional Chinese medicine. It is a 13-methyl-substituted derivative of berberine (BBR). BBR is well known as an eminent component in traditional Chinese and Ayurvedic medicine for more than 2000 years and is widely distributed in plant tissues. BBR has attracted much interest for its extensive pharmacological actions that have antibacterial, anti-inflammatory, antitumor, antiobesity, and hypercholesterolemic activities [12–14]. Recently, it was suggested that 13-MB has better performance than BBR in certain types of inflammatory diseases. The potential anti-inflammatory role of 13-MB has been reported in previous studies [14–16]. However, it is unclear whether 13-MB acts as an anti-inflammatory agent in atherosclerosis. Thus, we aimed to explore the role of 13-MB in H_2_O_2_-treated HUVECs, which is similar to vascular endothelial dysfunction in atherosclerosis. We attempted to confirm whether 13-MB improves endothelial dysfunction and whether it is related to the NLRP3 inflammasome and autophagy.

## Materials and methods

### Chemicals and reagents

13-Methylberberine (Cayman, Ann Arbor, Michigan, USA) was dissolved in dimethylsulfoxide (DMSO) to prepare a stock solution (20 mM), aliquoted and stored at − 20 °C. The Annexin V-FITC assay kit and CCK-8 assay kit were purchased from Beyotime (Shanghai, China). The DCFH-DA assay kit was purchased from BioVision (Shanghai, China). A mitochondrial membrane potential kit (JC-10 Assay) was obtained from Solarbio (Beijing, China). The following antibodies were used: rabbit anti-NLRP3, caspase-1, GAPDH, and anti-rabbit IgG (Cell Signaling Technology, Beverly, MA, USA). Western blot reagents, including enhanced chemiluminescence (ECL), were purchased from Amersham Biosciences (Piscataway, NJ, USA). ELISA kits were obtained from R&D Systems (Minneapolis, MN).

### Cell culture

HUVECs were obtained from the American Type Culture Collection (ATCC, Manassas, VA, USA) and cultured in high glucose DMEM (Dulbecco’s modified Eagle’s medium), supplemented with 10% FBS, 10 μg/mL penicillin, and 100 μg/mL streptomycin in an incubator at 37 °C with a humidified atmosphere of 5% CO_2_. HUVECs were used for our experiments within 6 months.

### Detection of cell viability by CCK-8 assay

A cell count kit-8 (CCK-8 Beyotime, China) assay was utilized to quantitatively evaluate cell viability. HUVECs were seeded onto 96-well culture plates and incubated for 24 h. After the cells reached 70–80% confluence, they were treated with 13-MB (1 μM) for 24 h, followed by hydrogen peroxide (100 μM) for another 6 h. Then, CCK-8 (10 μM) was added to each well and incubated at 37 °C for 2 h. The absorbances at 450 nm were determined by using a microplate reader (BioTek Instruments, VT, USA). DMEM containing 10% CCK-8 was used as a control.

### Determination of cell apoptosis by flow cytometry

Cell apoptosis was evaluated by flow cytometry with an Annexin V-FITC/PI dual staining detection kit (Beyotime, China). Briefly, HUVECs were seeded onto 6-well plates at a density of 1 × 10^6^ cells/well in DMEM supplemented with 10% FBS with or without 13-MB 1 μM for 24 h prior to treatment with H_2_O_2_ 100 μM for 6 h. After incubation, both floating and adherent cells were harvested, followed by Annexin V-FITC/PI double staining and analysis by flow cytometry (FCM; BD Bioscience).

### Measurement of intracellular ROS levels

2′,7′-Dichlorodihydrofluorescein diacetate (DCFH-DA) (BioVision, Shanghai, China) was used as an indicator of intracellular hydrogen peroxide in cells. HUVECs in a logarithmic growth phase (3 × 10^5^ cells in each well) were treated with various reagents as described above and then labeled with 2.5 µM DCFH-DA for 20 min. ROS levels were detected by using a microplate reader at excitation and emission wavelengths of 495 and 562 nm, respectively. Additionally, parallel-treated cells were collected, washed with cold PBS and analyzed by flow cytometry.

### Measurement of mitochondrial membrane potential

JC-10 was used to determine mitochondrial membrane potential (MMP) [17–19]. HUVECs were pretreated with 13-MB for 24 h with or without H_2_O_2_, an early-stage autophagy inhibitor (SAR405, 1 µM, pretreatment for 1 h), or a late-stage autophagy inhibitor (bafilomycin A_1_, BAF, 100 nM, pretreatment for 1 h). After treatment, the cells were washed with PBS and incubated with 5 µM JC-10 for 30 min in the dark. The fluorescence intensity for both J-aggregate and monomeric forms of JC-10 was measured by fluorescence microscopy. Red fluorescence was monitored using excitation and emission wavelengths of 585 and 590 nm, and green fluorescence was monitored using excitation and emission wavelengths of 515 and 529 nm. After subtracting the blank values, red/green fluorescence ratios per cell were calculated.

### Western blot analysis

After incubation with H_2_O_2_ with or without 13-MB pretreatment, HUVECs were collected and lysed using RIPA lysis buffer, followed by measurement of protein concentration using a BCA protein assay kit. The protein samples were separated by 12% SDS-PAGE and transferred to a PVDF membrane. Blots were blocked with 5% nonfat dry milk in TBST, followed by incubation with primary antibodies overnight at 4 °C and HRP-conjugated secondary antibodies at room temperature for 1 h. Enhanced chemiluminescence was used to detect protein signals. The intensities of the relative protein bands were quantified by Image Lab Software.

### ELISA analysis

HUVECs were treated as described above. IL-1β production in the supernatant of cells was measured with an ELISA kit according to the manufacturer’s instructions.

### Transfection and confocal microscopy

The PcDNA3.1-mRFP-EGFP-LC3 plasmid was transiently transfected into HUVECs using Lipofectamine 8000 transfection reagent (Beyotime, China) and Opti-MEM. Transfection was performed according to the manufacturer’s protocol. One day after transfection, the medium was changed. The cells were treated with 13-MB (0.1, 1, 10 µM), the autophagy inducer Torin 1 (200 nM), and the autophagy inhibitor BAF (100 nM) as positive controls. After 24 h of treatment, the cells were fixed with 4% paraformaldehyde (PFA) and examined using a confocal laser scanning microscope (FluoView fv1000, Olympus, Japan).

### Statistical analysis

All data are expressed as the mean ± standard deviation of at least three independent experiments. Statistical analysis was performed using GraphPad Prism 6 software, and statistical comparisons were made using one-way analysis of variance or Student’s *t*-test (unpaired). A value of *P* < 0.05 was considered statistically significant. *P < 0.05, **P < 0.01, ***P < 0.001, and ****P < 0.001.

## Results

### 13-MB alleviates H_2_O_2_-induced cytotoxicity in HUVECs

**Fig. 1:**
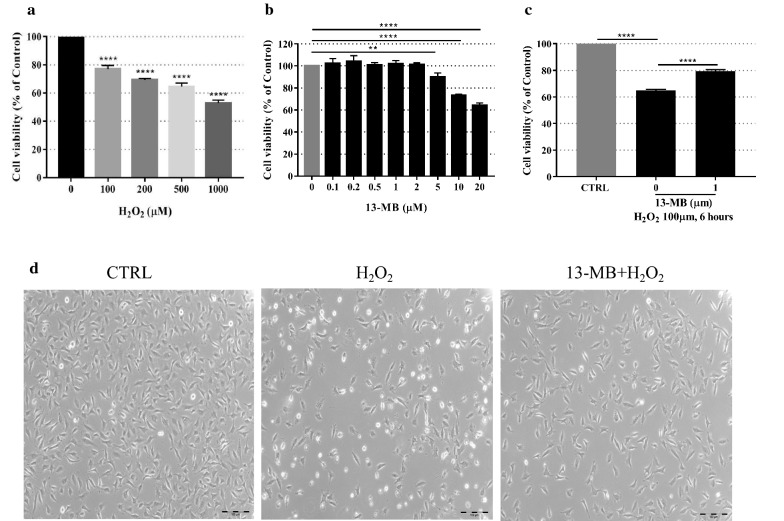
13-MB alleviates H_2_O_2_-induced cytotoxicity in HUVECs. **a** The HUVECs were incubated with various concentrations of H_2_O_2_ (0–1000 µM) for 24 h to choose an optimal oxidative stress condition. **b** The HUVECs were treated with an increasing concentration of 13-MB (0–10 µM) to exclude the toxic effects on cells. **c** The HUVECs were pretreated with 13-MB for 24 h followed by H_2_O_2_ for 6 h to assess the protective effect of 13-MB. All of them were determined by CCK-8 assay. ** and **** indicate a significant difference at the level of *P* < 0.01 and *P *< 0.001, respectively, compared to the control group. **d** The HUVECs were pretreated with 1 µM 13-MB before 100 µM H_2_O_2_. The cytoprotective effect of 13-MB in HUVECs was confirmed by morphological observations under bright field microscopy (×40 magnification). Scale bar = 100 µm

First, the cytotoxicity of H_2_O_2_ (0, 100, 200, 500 and 1000 µM) in HUVECs was determined by a CCK-8 assay. As shown in Fig. 1a, a dose-dependent increase in HUVECs was observed with H_2_O_2_ exposure. The optimal oxidative stress condition for cell injury was observed at 100 µM H_2_O_2_ treatment for 24 h; thus, this concentration was used for the subsequent experiments. The effects of 13-MB treatment alone on the cells was evaluated to exclude toxic effects on HUVECs. As shown in Fig. 1b, treatment with 13-MB at concentrations below 5 µM for 24 h did not influence cell survival. Furthermore, to assess the protective effect of 13-MB, HUVECs were pretreated with 1 µM for 24 h, followed by 6 h of treatment with 100 µM H_2_O_2_. The 13-MB pretreatment groups showed a significant improvement in cell viability. Therefore, we concluded that 13-MB protects HUVECs against H_2_O_2_-induced cell injury (Fig. 1c).

Additionally, the cytoprotective effect of 13-MB was confirmed by morphological observations under bright field microscopy. As shown in Fig. 1d, a decrease in the number of and morphological changes in cells were shown in H_2_O_2_-treated HUVECs, which were different from those of the control. However, the cell number and normal morphology of HUVECs with 13-MB pretreatment were almost unchanged. These data indicate that 13-MB prevents oxidative injury in HUVECs.

### 13-MB attenuates H_2_O_2_-induced apoptosis in HUVECs

**Fig. 2:**
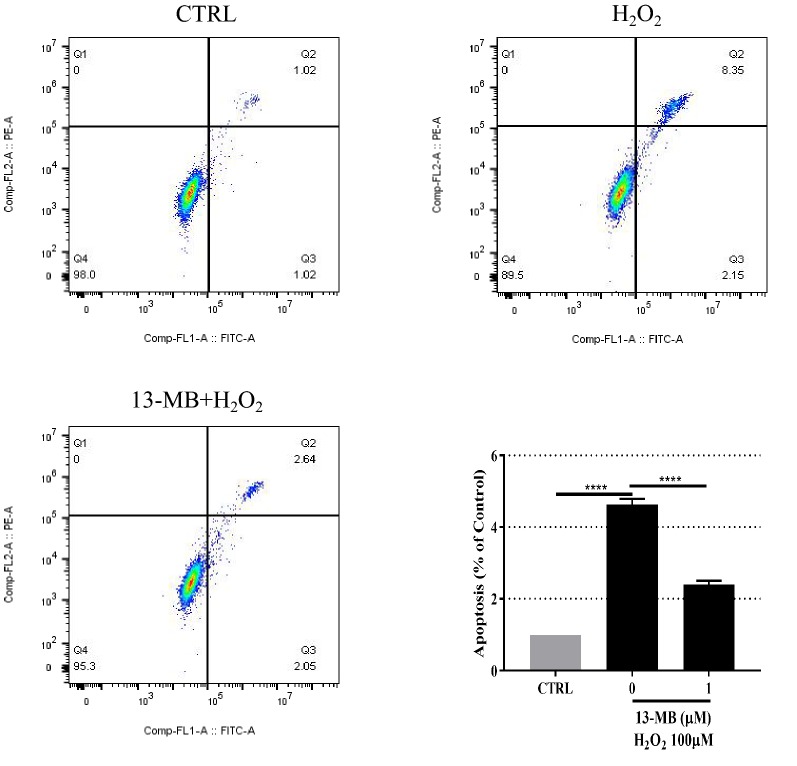
13-MB attenuates H_2_O_2_-induced apoptosis in HUVECs. HUVECs were pretreated with 1 µM 13-MB for 24 h prior to 100 µM H_2_O_2_ for 6 h. Then the cells were incubated with Annexin V-FITC/PI double staining. The rate of apoptosis was evaluated by flow cytometry. ****P < 0.001

To determine whether 13-MB attenuates H_2_O_2_-induced cell injury associated with apoptosis, HUVECs were pretreated with 1 µM 13-MB for 24 h prior to treatment with 100 µM H_2_O_2_ for 6 h. Flow cytometry analysis with Annexin V-FITC/PI double staining showed that apoptotic cells were significantly increased in the H_2_O_2_ treatment group compared with those of the control group, whereas the apoptotic rate decreased in response to 13-MB (Fig. 2). These results revealed that 13-MB prevented HUVECs from undergoing H_2_O_2_-induced apoptosis.

### 13-MB reduces H_2_O_2_-induced ROS generation in HUVECs

**Fig. 3:**
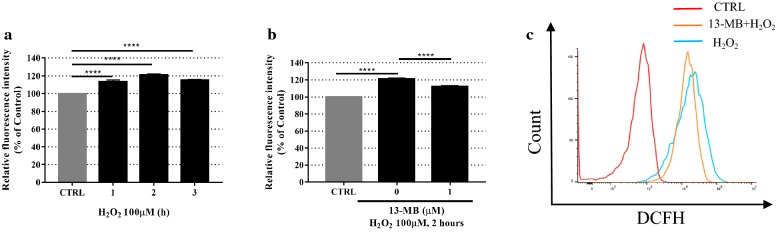
13-MB reduces H_2_O_2_-induced ROS generation in HUVECs. **a** HUVECs were exposed to 100 µM H_2_O_2_, then labeled with 2.5 µM DCFH-DA for 20 min, the fluorescence intensity of ROS was detected at 1 h, 2 h, 3 h. **b** Cells were pretreated with 1 µM 13-MB for 24 h followed by 100 µM H_2_O_2_ for 2 h, labeled with 2.5 µM DCFH-DA for 20 min. The levels of ROS were detected by the use of a microplate reader. **c** The parallel-treated cells were analyzed by flow cytometry. ****P < 0.001

To assess whether 13-MB reduced H_2_O_2_-induced ROS generation in HUVECs, cells were pretreated with 1 µM 13-MB for 24 h, followed by 100 µM H_2_O_2_ for 2 h. Then, the cells were incubated with 2.5 µM DCFH-DA. As shown in Fig. 3a, 100 µM H_2_O_2_ alone markedly increased ROS generation at 2 h. However, pretreatment with 13-MB downregulated ROS production, as measured by the fluorescence intensity in response to H_2_O_2_ (Fig. 3b). Moreover, flow cytometry analysis of DCFH-DA staining showed that 13-MB mitigated the increase in ROS generation in HUVECs exposed to H_2_O_2_, similar to the fluorescence intensity results (Fig. 3c). Taken together, 13-MB reduces excessive ROS production stimulated by H_2_O_2_, which was associated with oxidative damage.

### 13-MB inhibits NLRP3 inflammasome activation in HUVECs

**Fig. 4:**
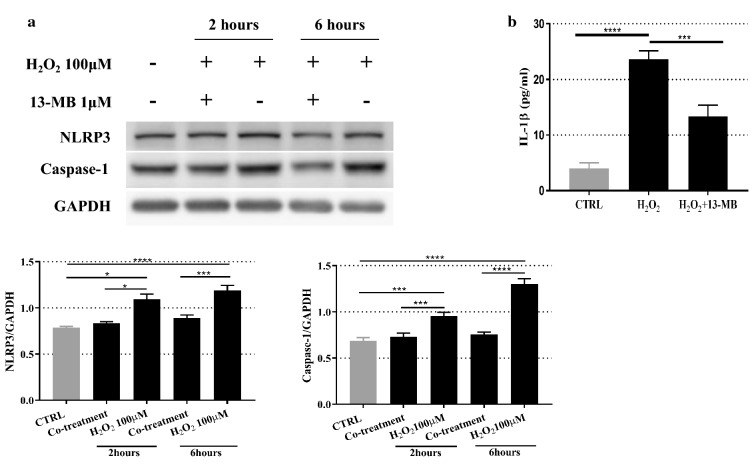
13-MB inhibits NLRP3 inflammasome activation in HUVECs. **a** The expression levels of NLRP3 and Caspase-1 in H_2_O_2_-treated HUVECs were measured by Western blotting. **b** The levels of IL-1β in supernatant of cells were measured by ELISA. The Western blotting results are representative of three different experiments. The ELISA data are expressed as the mean ± SD of three separate experiments. *** and **** *P* < 0.001

To confirm that 13-MB inhibits ROS-induced activation of the NLRP3 inflammasome in HUVECs, the protein expression levels of NLRP3 and Caspase-1 were measured by Western blotting. As expected, pretreatment with 1 µM 13-MB resulted in a reduction in NLRP3 and Caspase-1 protein levels in HUVECs under oxidative stress compared with those of H_2_O_2_-treated cells. Moreover, treatment with 100 µM H_2_O_2_ alone produced a significant increase in the protein levels of NLRP3 and Caspase-1 compared with those of the untreated cells (Fig. 4a).

The cytokine IL-1β, which is downstream of the NLRP3 inflammasome, acts as an important mediator for the development of atherosclerosis and was quantified by ELISA [20]. As shown in Fig. 4b, the level of IL-1β was significantly higher in the H_2_O_2_-treated group than in the control group, while 13-MB treatment markedly reduced the level of IL-1β compared to that of H_2_O_2_-treated cells.

These results showed that the NLRP3 inflammasome is directly activated when exposed to H_2_O_2_, which may influence the level IL-1β secretion in HUVECs. However, pretreatment with 13-MB suppressed NLRP3 inflammasome activation and downregulated the level of IL-1β.

### 13-MB inhibits the NLRP3 inflammasome by enhancing autophagy in HUVECs

**Fig. 5:**
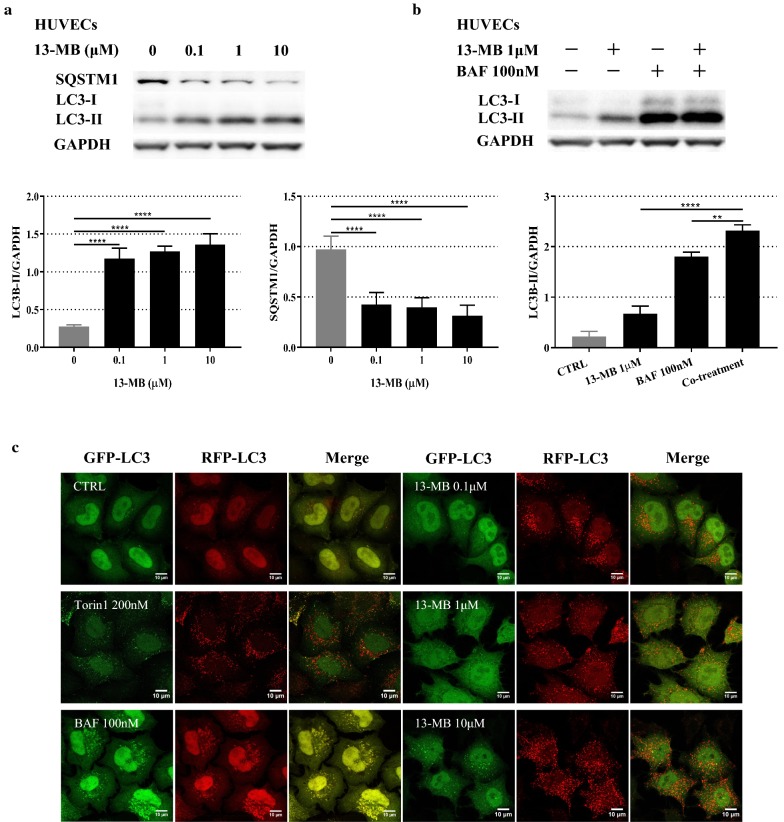
13-MB induces autophagy in HUVECs. **a** HUVECs were treated with 13-MB in a dose-dependent manner (0.1, 1, 10 µM). The protein expression levels of LC3-II/LC3-I and SQSTM1/P62 were measured by Western blotting. **b** HUVECs were treated with 1 µM 13-MB, 100 nM BAF, and pretreated with BAF for 1 h followed by 13-MB. The expression levels of LC3-II/LC3-I were measured by Western blotting. **c** 13-MB induces autophagy in HUVECs through autophagosome quantification with microscopic analysis. The quenching/degradation expressing of both GFP and RFP in HUVECs were examined by the use of a confocal laser scanning microscope. Scale bar = 10 µm

Autophagy is a cellular process of self-digestion that disassembles damaged proteins and organelles. An increasing number of studies have indicated that autophagy plays an important role in regulating NLRP3 inflammasome activation [21–23]. According to these findings, we investigated whether 13-MB modulates autophagy in HUVECs. The results reflected in autophagosome formation-related biochemical changes and autophagy substrate degradation [24]. We measured the ratio of LC3-II/LC3-I stimulated by 13-MB in HUVECs. The level of SQSTM1/p62 protein was used to measure autophagy substrates. The results showed that 13-MB increased LC3-I to LC3-II conversion and decreased SQSTM1/p62 levels in a concentration-dependent manner in HUVECs (Fig. 5a).

Moreover, compared with the levels induced by 13-MB or BAF treatment alone, cotreatment further increased the LC3-II protein level (Fig. 5b).

To further investigate the effect of 13-MB on autophagy induction, we transfected a plasmid into HUVECs and examined the quenching/degradation of both GFP and RFP in HUVECs using a confocal laser scanning microscope [25]. In this assay, much red puncta were present in cells when autophagy was induced. In contrast, it would give rise to both green and red signals, which appeared yellow, upon autophagy inhibition. Figure 5c shows that 13-MB modulates the RFP-GFP-LC3 distribution in HUVECs in a concentration-dependent manner. Treatment with 13-MB enhanced RFP-LC3 puncta formation and colocalization, which was similar to the effect of Torin 1 (a traditional autophagy inducer). Moreover, much RFP-LC3 puncta were distributed in HUVECs with increasing concentrations of 13-MB. However, both green and red signals appeared in HUVECs in the presence of BAF (a common autophagy inhibitor).

Thus, these data clearly provide evidence that 13-MB increased LC3-II conversion, decreased SQSTM1/p62 protein levels, and enhanced RFP-LC3 puncta formation in HUVECs, further confirming that 13-MB induces autophagy, thereby suppressing NLRP3 inflammasome activation in HUVECs.

### 13-MB preserves mitochondrial membrane potential by autophagy induction in HUVECs

**Fig. 6:**
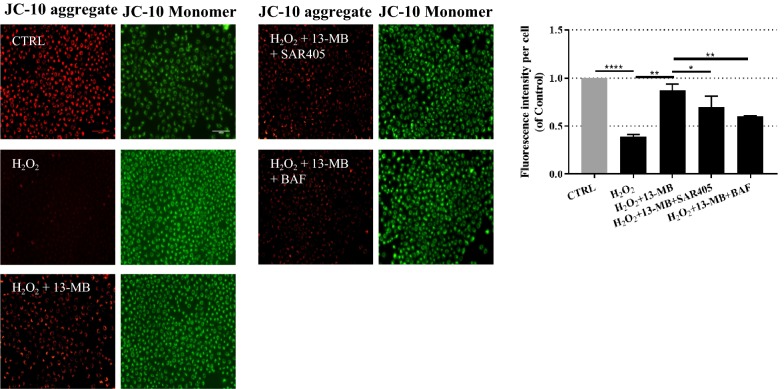
13-MB preserves mitochondrial membrane potential via autophagy induction in HUVECs. HUVECs were treated with 13-MB prior to H_2_O_2_, with the pretreatment of SAR405(1 μM) for 1 h, or pretreatment of BAF (100 nM) for 1 h. After that, the cells were incubated with JC-10 at 5 µM for 30 min in the dark. The fluorescent intensity for both aggregate and monomeric forms of JC-10 were measured with fluorescence microscopy. Red/green fluorescence ratios per cell were calculated. Scale bar = 100 µm. *P < 0.05, **P < 0.01, ***P < 0.001

Changes in mitochondrial membrane potential (MMP) can reflect the extracellular environment in cells [26]. A decrease in MMP is an indication of apoptosis caused by ROS in the early stage and reflects mitochondrial damage [27, 28]. Growing evidence has demonstrated that impaired mitochondria induce autophagy. Most studies using drugs that target mitochondria have shown that NLRP3 inflammasome activation and IL-1β secretion are not only dependent on mitochondrial ROS generation but also sensitive to mitochondrial functional disturbances [29]. Therefore, to confirm the protective effect of 13-MB on mitochondrial-mediated autophagy, JC-10 dye was used to measure MMP. In heathy cells with high MMP, JC-10 spontaneously forms complexes known as JC-10 aggregates with intense red fluorescence. In contrast, in apoptotic or unhealthy cells with low MMP, JC-10 remains in the monomeric form, which shows only green fluorescence. As shown in Fig. 6, the H_2_O_2_-treated group exhibited an increasing number of JC-10 monomers compared with that of the control group, suggesting a decrease in MMP. Conversely, 13-MB treatment increased the number of JC-10 aggregates, which indicated that the MMP was preserved. However, the addition of SAR405 (an early-stage autophagy inhibitor) and BAF (a late-stage autophagy inhibitor) attenuated the protective effect of 13-MB. These results indicated that ROS exacerbated mitochondrial damage. However, 13-MB showed a protective effect in maintaining MMP. Furthermore, autophagy played a pivotal role in 13-MB-mediated preservation of MMP.

## Discussion

In the present study, we showed that 13-MB protects HUVECs from H_2_O_2_-induced oxidative toxicity and apoptosis in the early stage. Moreover, 13-MB inhibited NLRP3 inflammasome activation and induced autophagy, as evidenced by CCK-8 assay, DCFH-DA assay, ROS measurement, MMP detection, Western blotting, ELISA, and laser scanning confocal microscopy. These in vitro functional experiments suggest that 13-MB improves endothelial dysfunction by inhibiting NLRP3 inflammasome activation via autophagy induction. These results suggest the possibility of using 13-MB as an autophagy inducer for treating NLRP3 inflammasome-associated diseases.

The role of vascular inflammation in atherosclerosis is well recognized by the immune system, which is stimulated by harmful factors, such as injury, pathogens and oxidative stress. Atherosclerosis is accompanied by a chronic, low-level inflammatory response that attracts innate and adaptive immune cells into the atherosclerotic plaque [30]. Attenuating the inflammatory response represents one of the fundamental therapeutic strategies against atherosclerosis available today. Natural products may still be the most abundant sources for new drug development [31]. BBR is well known for its naturally derived anti-inflammatory properties [32]. 13-MB, a newly synthesized, 13-methyl-substituted derivative of BBR, has better anti-inflammatory activity than BBR. However, 13-MB has not yet been investigated as a potential therapeutic agent against atherosclerosis. Increasing evidence suggests that oxidative stress contributes to cellular damage and appears to be a common apoptotic mediator. In our study, a model of H_2_O_2_-impaired endothelial cells was utilized to mimic the oxidative endothelial injury that occurs during the early stage of atherogenesis. Here, we showed that 13-MB controlled acute vascular inflammation by alleviating cytotoxicity and apoptosis induced by H_2_O_2_ in HUVECs. Moreover, 13-MB reduced ROS generation in H_2_O_2_-treated HUVECs. This study indicates the potential role of 13-MB in maintaining endothelial homeostasis.

The NLRP3 inflammasome is a multiprotein complex that plays a critical role in the innate immune system. It is activated by endogenous or exogenous stimuli and is involved in the process of sterile inflammation. Many clinical and experimental studies have reported that the NLRP3 inflammasome contributes to endothelial dysfunction in atherosclerosis. Therefore, inhibiting the NLRP3 inflammasome is a potential approach for atherosclerosis therapy. It has been reported that the inflammasome Nlrp3-caspase-1-IL-1β pathway triggers vascular endothelial inflammation in the progression of atherosclerosis [4]. In this situation, NLRP3 inflammasome formation requires a priming step that is provided by pattern recognition or cytokine receptors. Once activated, it induces Nlrp3 itself, leading to the recruitment and activation of caspase-1, and then caspase-1 cleaves pro-IL-1β into its mature bioactive form. Previous studies also showed that genetic loss of the inflammasome components Nlrp3 [33], caspase-1 [34], and IL-1β [35] leads to a reduction in atherosclerotic lesions. In our study, 13-MB suppressed the activation of inflammasome components, which decreased Nlrp3 expression, caspase-1 formation and IL-1β secretion. 13-MB negatively modulates the NLRP3 inflammasome and may be a therapeutic target for atherosclerosis.

Autophagy promotes cell survival by removing injured organelles and intracellular pathogens. Accumulating evidence indicates that autophagy, an intracellular degradation system that maintains cellular homeostasis, downregulates NLRP3 inflammasome activation. Autophagy captures and degrades the assembled complex of the NLRP3 inflammasome via ubiquitination and modulates its activity [36, 37]. Alterations in autophagic flux are implicated in atherosclerosis [38]. Consistent with these findings, Western blotting data and laser scanning confocal microscopy confirmed that autophagy was induced in HUVECs by 13-MB. The results in Fig. 5A show that 13-MB upregulated the accumulation of LC3-II and decreased SQSTM1/p62 levels. LC3-II and SQSTM1/p62 are two well-known markers of autophagy. An increased ratio of LC3-II/GAPDH is a marker of increasing autophagosomes, whereas SQSTM1/p62 is inversely correlated with autophagic flux [39]. Furthermore, an LC3 turnover assay was conducted to measure autophagic flux, which is based on the observation that LC3-II is degraded in autolysosomes. The protective effect of 13-MB was accompanied by LC3-II accumulation, as demonstrated by the use of an autophagy inhibitor, BAF, thereby indicating the upregulation of autophagy (Fig. 5b). Autophagic flux was morphologically traced with a mRFP-GFP-LC3 tandem construct in HUVECs, which confirmed the role of 13-MB as an autophagy inducer (Fig. 6). These findings revealed that 13-MB improved endothelial dysfunction through autophagy induction. It is possible that 13-MB promoted protective autophagy in endothelial dysfunction by inhibiting the NLRP3 inflammasome. However, the mechanism by which 13-MB inhibits NLRP3 inflammasome activation via autophagy induction in HUVECs requires further study.

**Fig. 7:**
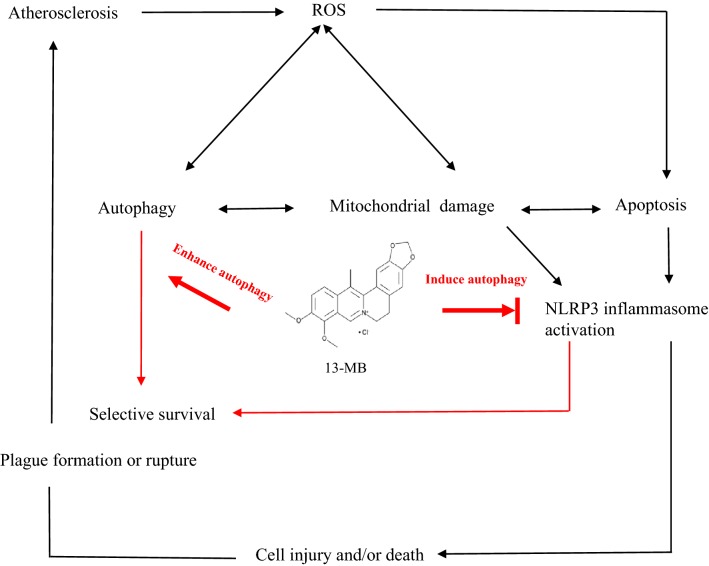
The pathway involved in 13-MB improving endothelial dysfunction by inhibiting NLRP3 inflammasome activation via autophagy induction in HUVECs

Considerable evidence suggests that mitochondria are the central organelles in autophagy and inflammatory responses. Conditions of oxidative stress markedly induce ROS production, which causes mitochondrial damage [40], resulting in endothelial cell injury and/or death and atheromatous plaque formation or rupture, which together lead to organ injury and high mortality in the host. It has been reported that autophagy negatively regulates NLRP3 inflammasome activation through many types of stimuli, including mitochondrial damage [41]. Decreased MMP is an indication of apoptosis caused by ROS in the early stage and reflects mitochondrial damage. Our analysis measuring MMP showed that 13-MB exerts a protective effect against mitochondrial damage by maintaining MMP. However, the addition of SAR405 (an early-stage autophagy inhibitor) and BAF (a late-stage autophagy inhibitor) attenuated the protective effect of 13-MB. Taken together, our study indicates that 13-MB affects endothelial cells, at least in part, through autophagy induction to maintain endothelial homeostasis. Furthermore, our data suggest that 13-MB attenuates endothelial dysfunction by inhibiting NLRP3 inflammasome activation via autophagy induction (Fig. 7).

However, there are some limitations in the present study. The detailed mechanism of autophagy induction by 13-MB to inhibit NLRP3 inflammasome activation is not well understood. Further study is needed to clarify this issue. In addition, we should further confirm the efficacy of 13-MB in vivo. An animal model should be established using transgenic animals to perform future atherosclerosis research. Therefore, further investigations of 13-MB in animal experiments are required to elucidate the role of 13-MB in the development of atherosclerosis.

## Conclusion

Our study reveals that 13-MB reverses H_2_O_2_-induced HUVEC injury. The underlying mechanisms by which 13-MB improves endothelial dysfunction were shown to involve cytoprotection, inhibition of NLRP3 inflammasome activation and enhanced autophagy. These results provide evidence that 13-MB has the potential to be a novel autophagy inducer for the prevention or treatment of NLRP3 inflammasome-associated inflammatory diseases, including atherosclerosis.

## Data Availability

The datasets used in this study are available from the corresponding author upon reasonable request.

## Abbreviations

13-MB: 13-methylberberine
BBR: berberine
ASCVD: atherosclerotic cardiovascular disease
HUVECs: human umbilical vein endothelial cells
ROS: reactive oxygen species
DMSO: dimethylsulfoxide
DMEM: Dulbecco’s modified Eagle’s medium
DCFH-DA: 2′,7′-dichlorodihydrofluorescein diacetate
PFA: paraformaldehyde
CCK-8: cell count kit-8
ECL: enhanced chemiluminescence
MMP: mitochondrial membrane potential
BAF: bafilomycin A_1_
ox-LDL: oxidized low-density lipoprotein
